# Biomechanical study of strength and stiffness of the knee anterolateral ligament

**DOI:** 10.1186/s12891-016-1052-5

**Published:** 2016-04-30

**Authors:** Camilo Partezani Helito, Marcelo Batista Bonadio, João Stefan Rozas, João Marcelo Pedroso Wey, Cesar Augusto Martins Pereira, Tulio Pereira Cardoso, José Ricardo Pécora, Gilberto Luis Camanho, Marco Kawamura Demange

**Affiliations:** Knee Surgery Division, Department of Orthopaedics and Traumatology, Institute of Orthopedics and Traumatology–Hospital and Clinics, Faculty of Medicine, University of São Paulo (IOT-HCFMUSP), São Paulo, Brazil; Biomechanical Laboratory Division, Department of Orthopaedics and Traumatology, Institute of Orthopedics and Traumatology–Hospital and Clinics, Faculty of Medicine, University of São Paulo (IOT-HCFMUSP), São Paulo, Brazil; Department of Orthopaedic Surgery, Catholic University of São Paulo, Sorocaba, São Paulo Brazil; Rua Dr. Ovídio Pires de Campos, 333, Cerqueira Cesar, São Paulo SP CEP: 05403-010 Brazil

**Keywords:** Knee, Anterolateral ligament, Biomechanics, Anatomy

## Abstract

**Background:**

Recent studies clearly characterize the anatomical parameters of the knee anterolateral ligament (ALL). The potential clinical importance of this ligament is exemplified by some patients with possible combined Anterior Cruciate Ligament (ACL) and ALL rupture who do not progress satisfactorily following isolated ACL reconstruction. Previous biomechanical studies have assessed the resistance parameters of the ALL in order to address potential reconstruction strategies; however, these have reported conflicting results. Thus, this study aimed to evaluate the linear resistance of the ALL by means of a biomechanical study in cadaveric knees.

**Methods:**

Fourteen cadaveric knees were used. The ALL was dissected, and all structures that connect the femur and the tibia, except for the ALL, were sectioned. The ALL was subjected to a tensile test with the knee around 30 to 40 degrees, in a way that the ALL was aligned with the machine. The strength at the maximum resistance limit, deformation and stiffness of the ALL were evaluated.

**Results:**

The mean maximum strength of the ALL was 204.8 +/- 114.9 N. The stiffness was 41.9 +/- 25.7 N/mm and the deformation 10.3 +/- 3.5 mm.

**Conclusion:**

The ALL has a mean ultimate tensile strength of 204.8 N. This suggests that simple bands of all autologous or homologous grafts commonly used in clinical practice for ligament reconstruction around the knee possess the required biomechanical resistance characteristics for ALL reconstruction.

## Background

Recent studies have characterized the anterolateral ligament (ALL) of the knee in detail [1–6]. This structure is regarded as having a complementary action to the anterior cruciate ligament (ACL) with regard to anterolateral rotational knee stability. Its potential clinical importance is exemplified by patients with possible combined ACL and ALL rupture who do not progress satisfactorily following isolated ACL reconstruction [7, 8].

Anatomical and histological studies have confirmed the presence of the ALL and showed well-organized dense connective tissue in the ALL substance, compatible with true ligament tissue. These findings have been corroborated by imaging studies of the ALL [4, 6, 9, 10]. Despite some controversy regarding femoral attachment, which has already been defined as anterior and distal or posterior and proximal to the Lateral Collateral Ligament (LCL), the tibial attachment between Gerdy’s tubercle and the fibular head is constant. Similar to the medial side, a meniscal attachment at the transition between the anterior horn and the meniscus body has been found [3, 4, 6, 11]. Radiographic landmarks and length change patterns of the ligament during flexion-extension were also studied [12–14].

Biomechanical studies that tested the anterolateral capsule and indirectly demonstrated the importance of the ALL to rotatory knee stability were conducted, and Zens et al. and Kennedy et al. tested the tensile properties of this ligament [15–18]. Despite using similar methods, these two biomechanical studies reported substantially different resistance characteristics of the ALL. This poses difficulty in creating appropriate reconstruction techniques that rely on such studies to define both the graft and fixation method to be used [17, 18].

Knowledge of the ligament’s tensile properties may contribute to a better understanding of the ligament’s behavior, allowing for a fact-based assessment of its contribution to knee stability. This is essential for the selection of suitable transplant and reconstruction techniques. Thus, the present study aimed to evaluate the linear resistance of the ALL by means of biomechanical tests in cadaveric knees.

## Methods

For this study, 14 unpaired knees from male fresh-frozen cadavers were used. The mean age was 62.6 +/- 8.38 (range from 49 to 77). Before testing, the specimens were thawed for 24 h. All tests were performed at room temperature and the specimens were constantly kept moist with saline solution. The study was conducted following approval from the ethics committee at our institution. Consent was given either during life or from the next of kin following death for the use of cadavers in scientific research.

The ALL was dissected in a standard manner from all cadavers used. The protocol used for dissection has already been described in previous studies [2–4]. Initially, the skin and subcutaneous tissue were dissected, followed by tenotomy of the quadriceps tendon in its myotendinous junction, medial parapatellar opening of the retinaculum and osteotomy of the anterior tibial tuberosity to access the anterolateral region of the knee without violating adjacent extra-articular soft tissue. The retropatellar fatpad was partially removed in order to create better vision. The entire iliotibial tract was cut approximately 5 cm proximal to the lateral epicondyle and then reflected and detached from Gerdy’s tubercle and surrounding areas. The biceps tendon was cut immediately proximal to the fibular head. Biceps expansions to the tibia were also carefully removed. The popliteus muscle tendon (PT) and the LCL were carefully isolated so as not to reach the ALL attachment on the lateral epicondyle.

**Fig. 1:**
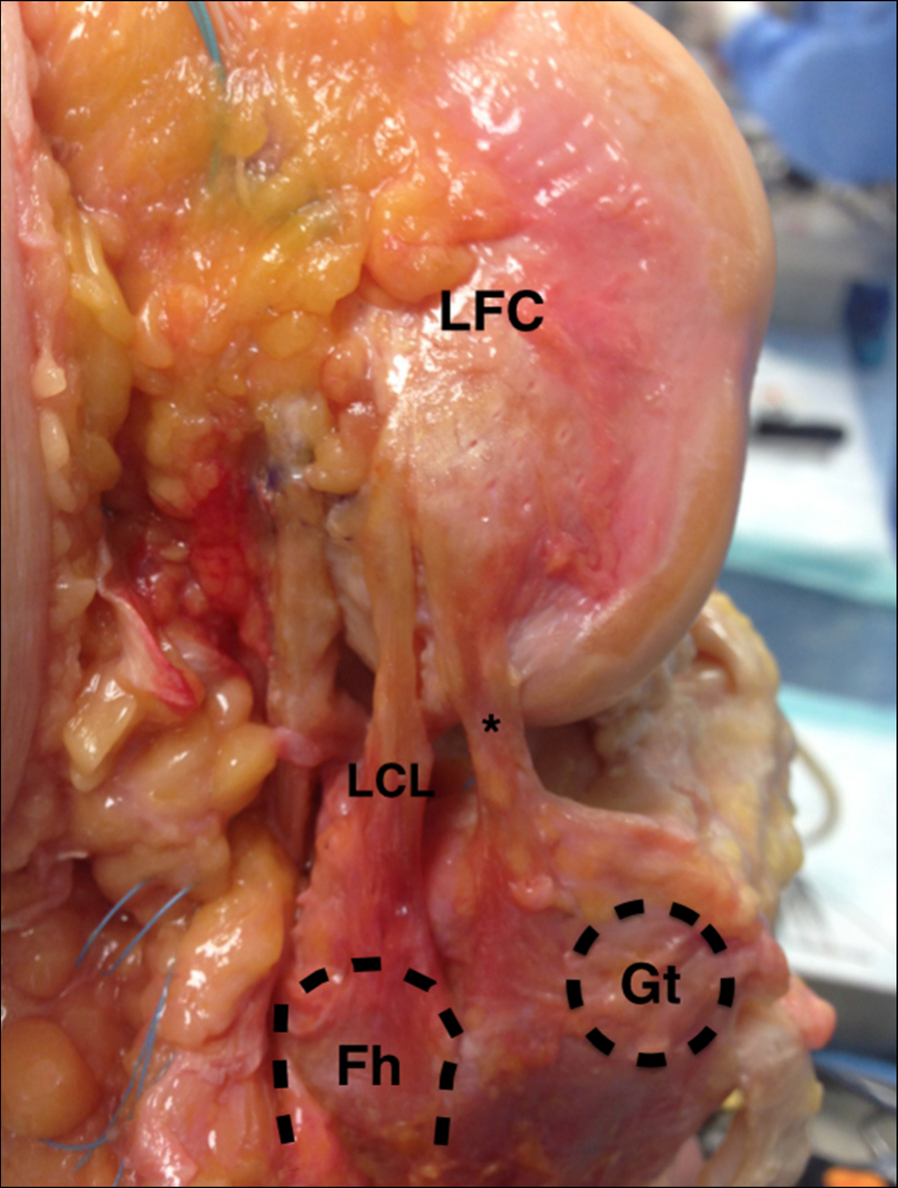
Lateral view of a right knee showing the anatomical features of the knee anterolateral ligament (asterisk). LFC – Lateral Femoral Condyle, LCL – Lateral Colateral Ligament, Fh – Fibular head, Gt – Gerdy’s tubercle

After these structures were isolated, capsular thickening could be clearly observed in the anterolateral region of the knee, which is consistent with the ALL, especially when performing flexion and internal rotation of the tibia. Starting at the femoral origin, the dissection was performed from the proximal to the distal region until the tibial insertion was isolated (Fig. 1).

After isolating the ALL, all intra-articular and peripheral structures of the knee were sectioned, and only the ALL remained intact on the lateral portion of the knee, which was the only remaining structure connecting the femur and the tibia.

The ligaments were subjected to a tensile test using a Kratos 5002 universal biomechanical test machine (Kratos Dynamômetros, São Paulo, Brazil), with a 100-kgf load cell, adjusted in a 20 kgf scale, with a crosshead speed of 20 mm/min. The force and deformation parameters from the testing machine were recorded on the computer through a system of ADS 2000 data acquisition 14 -bit resolution (Lynx Electronic Technology Ltda, São Paulo, Brazil) and a program written in Delphi 2006 (Borland software Corporation, Austin - Texas, USA), using the acquisition routines provided by the manufacturer of the acquisition system.

**Fig. 2:**
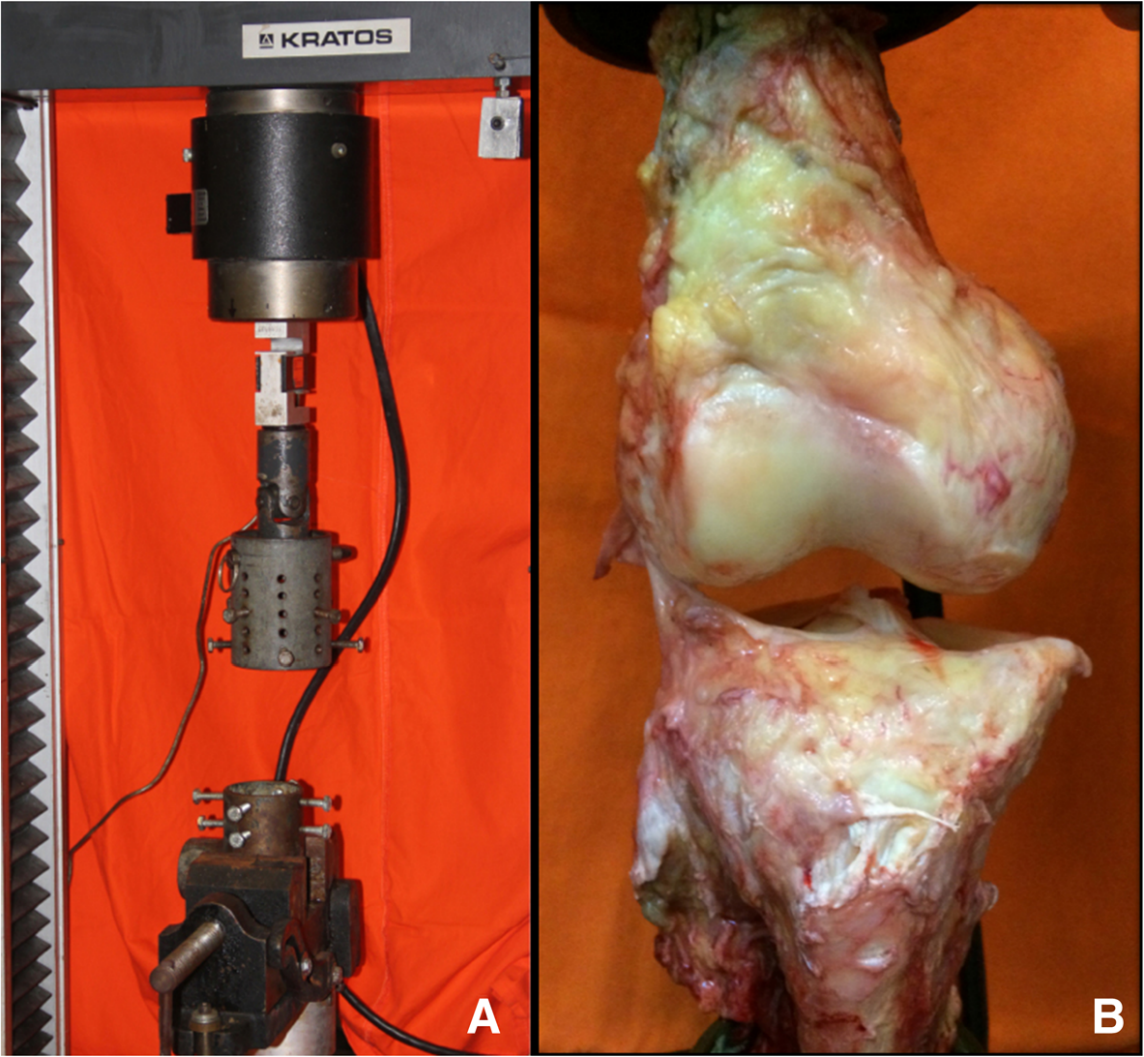
Picture showing the Kratos machine used to perform the biomechanical tests (**a**) and the dissected knee with only the Anterolateral Ligament connecting the femur to the tibia being tested (**b**)

The bone portions of the knee were connected to the machine with two tubular clips with radial screws, with one clip fixing the femur and one clip fixing the tibia. The femur was kept in the proximal region and the tibia in the distal region of the assembly, maintaining the tibial axis visually in alignment with the machine axis. In the assembly, the tibial tubular clip was fixed at the base of the machine using a bench vise, allowing only proximal displacement of the femur, which was attached to the moving part of the machine. The test was performed with the knee in approximately 30 to 40 degrees of flexion, such that the ALL was aligned with the machine (Fig. 2).

**Fig. 3:**
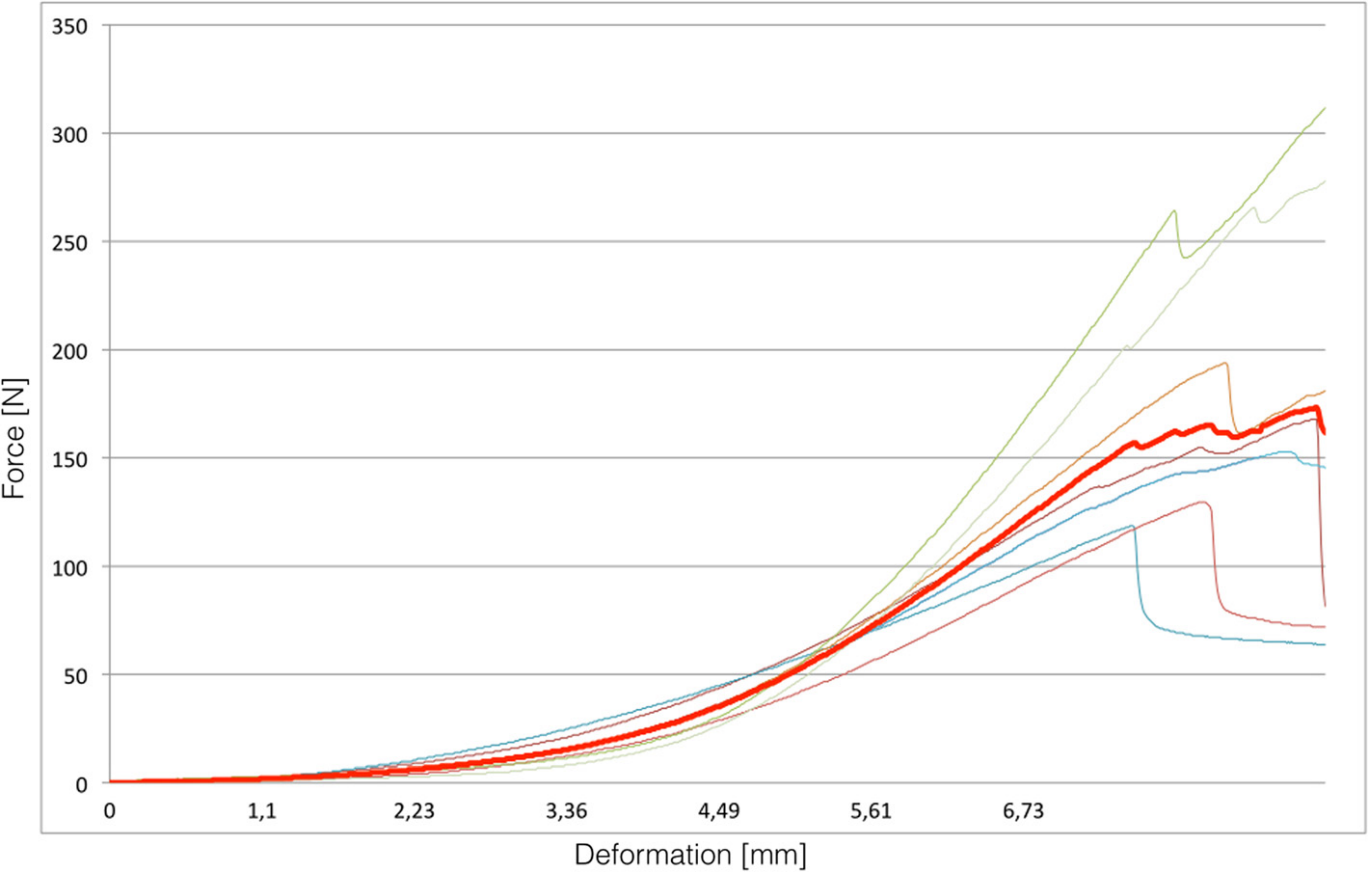
Graphic showing strength (N) and deformation (mm) examples of the knee anterolateral ligament (the red line is the average of studied knees). Not all samples are showed in the graphic

The parameters analyzed were strength at maximum ligament resistance limit, expressed in Newtons, and stiffness, defined as the ratio of the differences of strength and deformation between two points in the linear region of the strength versus deformation diagram, expressed in N/mm.

## Results

The ALL was found in all knee dissections. Its circular shape femoral attachment was anterior and distal to the LCL main attachment in 12 cases and immediately anterior in two cases. As the main insertion of the LCL is posterior and proximal to the lateral epicondyle as described by LaPrade et al., the ALL insertion was close to the lateral epicondyle center [19]. The band-shaped tibial attachment was found between Gerdy’s tubercle and the fibular head, around 5 to 10 mm distal to the lateral plateau.

The mean maximum strength of the ALL in the cases studied was 204.8 +/- 114.9 N (Fig. 3). The mean stiffness was 41.9 +/- 25.7 N/mm (Table 1).

**Table 1:** Knee data and Anterolateral Ligament biomechanical properties and failure mechanisms

Knee	Age (years)	Load to failure (N)	Stiffness (N/mm)	Mechanism of failure
1	54	168.04	37.96	Midsubstance tears
2	71	114.60	23.68	Midsubstance tears
3	56	327.12	36.62	Midsubstance tears
4	68	118.57	25.25	Midsubstance tears
5	54	193.77	45.38	Tibial detachment
6	71	129.62	32.59	Midsubstance tears
7	56	264.17	85.51	Midsubstance tears
8	61	152.93	34.20	Midsubstance tears
9	62	384.30	96.76	Midsubstance tears
10	68	363.88	47.44	Midsubstance tears
11	59	49.38	14.41	Femoral detachment
12	77	145.72	18.08	Segond fracture
13	71	371.70	71.58	Midsubstance tears
14	49	83.01	17.26	Femoral detachment
Average	62.6	204.8	41.9	
Median	61.5	160.5	35.4	
Standard Deviation	8.4	114.9	25.7	
Maximun	77	384.30	96.76	
Minimun	49	49.38	14.41	

In 10 knees, ligament failure was caused by stretching of the ligament body fibers, or midsubstance tears. In two cases the ALL was detached from its femoral origin and in one case it was detached from its tibial origin. Finally, in one case a distal tibia attachment avulsion fracture (“Segond fracture”) occurred. There were no cases of femoral avulsion fractures. The deformation of ALL at the breaking point was 10.3 +/- 3.5 mm, around 30 % of the ligament total length.

## Discussion

In the present study, the ALL had a maximum mean strength of 204.8 N and a mean stiffness of 41.9 N/mm. These findings are important because they enable the selection of adequate grafts and fixation methods for possible reconstructions of the ALL associated with reconstructions of the ACL.

Although there are no specific indications in the literature for ALL reconstruction, several authors have published suggestions for extra-articular reconstruction associated with intra-articular ACL reconstruction. Revision cases or athletes with high pivot-shift scores on the preoperative assessment are possible candidates [20–26]. The ALL reconstruction would involve an extra-articular reconstruction technique, but respecting the anatomical parameters of this structure in the anterolateral region of the knee [1–6]. Some classical extra-articular reconstruction procedures reroute a strip of the iliotibial tract deep to the LCL in order to reconstruct the ACL. However, due to the extra-articular position, these reconstructions merely focus on controlling internal tibial rotation and only have limited capacity reducing anterior tibial translation [24].

According to the values measured, the ALL exhibits maximum strength values similar to those of the medial patellofemoral ligament (MPFL) and lower than those of other medial and lateral peripheral ligaments of the knee [27–30]. According to studies by Mountney et al. and Herbort et al., the MPFL had a maximum strength of 208 N and 190.7 N, respectively [28, 29]. Wijdicks et al. reported a strength of 557 N for the superficial medial collateral ligament, and Ciccone II et al. reported a maximum strength of 460 N for the LCL [27, 30]. According to our measured values, the ALL has maximum strength values lower than half of these collateral structures, which suggests that the ALL may not be the only important structure in restraining rotational knee laxity and is probably associated with the ACL and other lateral structures to perform this role [31].

The present study found a similar maximum strength of the ALL compared to Kennedy et al. [18], and a higher mean maximum strength compared to Zens et al. (50 N) [17]. Zens et al. found interligamentous tears in four cases, while Kennedy et al. observed four midsubstance tears, six Segond fractures, four femoral tears at the femoral origin and one tear at tibial origin. Most of our cases failed at the ALL body, similar to the results found by Zens et al., but we had four variations. The cases in which the failure happened as a femoral detachment presented the lower stress values (49 and 83 N), well below the average found. None of the studies showed a femoral avulsion fracture. Even though the specimens ages differed among the three studies (Zens et al – 86.5 years, Kennedy et al – 58.2 years, current study – 62.6), the differences in maximum load at failure found cannot be credited to this fact, as suggested by Zens et al. This study found a higher age and a higher value for the ALL strength than Kennedy et al. A possible explanation for the lower results in the study by Zens at al. may be due to weakening of the ligament during dissection in order to completely isolate it.

Regarding grafts that could be used in possible reconstructions of this structure, based on the ALL strength and according to biomechanical tests performed by Pearsall IV et al. and Noyes et al. [32, 33], simple semitendinosus (1216 N), simple gracilis (838 N), a strip of the iliotibial tract, or even anterior tibial, posterior tibial or long peroneal tissue bank grafts would be suitable. The possibility of using only the simple semitendinosus or the simple gracilis would facilitate a combined reconstruction of the ALL and the ACL. The ACL graft could be composed of the triple semitendinosus with a simple gracilis, for example. The remaining portion of the gracilis would then be used for the ALL reconstruction [34, 35]. Similar to the MPFL, there is no available graft for reconstruction which exactly mimics the tensile properties of the ALL. Thus, it seems important to pay close attention to positioning and tensioning of the graft in order to avoid over-constraining of the lateral compartment [31, 36]. Even with combined ALL reconstruction, studies have shown that the ACL diameter should be at least 8 mm to minimize failures in the intra-articular reconstruction [37].

Regarding graft fixation, biomechanical tests are still necessary to evaluate which technique is best for the repair or reconstruction of the ALL. Considering it shows strength values similar to those of the MPFL, it is possible to infer that ALL fixation using anchors will produce suitable functional clinical results. However, according to biomechanical studies, fixation with interference screws is more resistant [38]. The use of anchors for fixation would avoid creating one more bone tunnel in addition to the ACL tunnel, which would avoid the convergence problem that exists, for example, in combined reconstructions of the ACL and the posterolateral corner structures [39, 40]. Furthermore, because of the anatomical proximity between the ALL and the LCL, a lateral tunnel at the anatomical point of the ALL could cause an iatrogenic injury of the femoral insertion site of the LCL [1–6, 41].

Despite some differences in descriptions and controversies in the current literature [42–44], Claes et al. [42] suggested the Segond fracture as a result of an avulsion of the ALL in a study using anatomy and magnetic resonance imaging. Kennedy et al., using similar methodology, found most of the failures occurred because of a Segond fracture [18]. In the present study, we could reproduce only one case of failure due to tibial bone avulsion of the ALL. This may have occurred because of the linear tensile testing setup rather than a real life ACL injury.

This study is important because it characterizes the biomechanical resistance of the ALL. Despite the findings described, there is no clear indication for ALL reconstruction because neither its biomechanical importance to rotational stability nor its healing potential after injury is fully defined.

This study presented some limitations, such as the number of cadavers used for biomechanical testing, the fact that we only tested the ALL with an axial tensile testing setup and neutral rotation, the use of only male cadaver knees and the advanced age of the knees studied. We are also assuming that the fresh-frozen cadavers utilized in this study exhibit similiar biomechanical properties as tissue in vivo. Due to the ALL connections to the LCL and adjacent structures, it is possible that in some cases a small portion of the ALL could also be removed, which may alter its biomechanical characteristics.

## Conclusions

The ALL has a mean ultimate tensile strength of 204.8 N. This suggests that simple bands of all autologous or homologous grafts commonly used in clinical practice for ligament reconstruction around the knee possess the required biomechanical resistance characteristics for ALL reconstruction.

### Ethics and consent to participate

The study was approved by the scientific committee of the Institute of Orthopedics and Traumatology, University of São Paulo, Brazil.

### Consent to publish

Not applicable.

### Availability of data and materials

All the data that supports this study is contained within the manuscript. Requests for further detail on the dataset and queries relating to data sharing arrangements may be submitted to the corresponding author.

## Abbreviations

ACL: anterior cruciate ligament
ALL: anterolateral ligament
LCL: lateral collateral ligament
MPFL: medial patellofemoral ligament
PT: popliteus muscle tendon
